# Tau accumulation in the nucleus accumbens in tangle-predominant dementia

**DOI:** 10.1186/2051-5960-2-40

**Published:** 2014-04-08

**Authors:** Ito Kawakami, Masato Hasegawa, Tetsuaki Arai, Kenji Ikeda, Kenichi Oshima, Kazuhiro Niizato, Naoya Aoki, Katsuse Omi, Shinji Higashi, Masato Hosokawa, Yoshio Hirayasu, Haruhiko Akiyama

**Affiliations:** 1 Dementia Research Project, Tokyo Metropolitan Institute of Medical Science, 2-1-6 Kamikitazawa, Setagaya-ku, Tokyo 156-8506, Japan; 2 Department of Psychiatry, Graduate school of Medicine, Yokohama City University School of Medicine, Yokohama, Japan; 3 Department of Neuropathology and Cell Biology, Tokyo Metropolitan Institute of Medical Science, Tokyo, Japan; 4 Department of Psychiatry, Graduate School of Comprehensive Human Sciences, University of Tsukuba, Tsukuba, Japan; 5 Department of inflammation of Pathology, Faculty of Medicine, Kagawa University, Takamatsu, Japan; 6 Tokyo Metropolitan Matsuzawa Hospital, Tokyo, Japan

**Keywords:** Neurofibrillary tangle, Alzheimer disease, Propagation, Delusion

## Abstract

**Background:**

Tangle-predominant dementia (TPD) is characterized neuropathologically by numerous neurofibrillary tangles in the limbic areas with no or occasional senile plaques throughout the brain. TPD is an under-recognized disease, while it is a common cause of dementia in those over 80 years of age. In the present study, we describe hyperphosphorylated tau (tau) accumulation in the nucleus accumbens (Acb) in patients with TPD.

**Results:**

We investigated immunohistochemically the brain tissues from 7 patients with TPD, 22 with Alzheimer disease (AD) and 11 non-demented aged subjects. In the Acb of all 7 TPD patients, a considerable number of tau positive neurons were found together with many neuropil threads. The tau deposits in the Acb were labeled with all the anti-tau antibodies used in the present study. They included conformational change-specific, phosphorylation-specific and phosphorylation-independent antibodies. The Acb consists of the predominant medium-sized neurons with a small number of large neurons. Both the cell types were affected by tau pathology in TPD. Tau accumulation in the majority of such neurons appeared to be pretangle-like, diffuse deposits with only occasional paired helical filament formation. Tau positive neurons were also found in the Acb in some AD and non-demented aged subjects but much fewer in the majority of cases. The immunoblot analyses of fresh frozen samples of the Acb and parahippocampal cortex from 3 TPD and 3 AD patients revealed that the insoluble tau in the Acb was a mixture of the 3- and 4-repeat isoforms.

**Conclusions:**

To our knowledge, this is the first report on the occurrence of tau accumulation in the Acb in TPD. The Acb receives direct and massive projections from the hippocampal CA1 and subiculum where neurofibrillary tangles are known to occur more frequently in TPD than in AD. The prevalence of abnormal tau accumulation in the Acb in TPD may support the idea that abnormal tau aggregation propagates via neural circuits. In all but one TPD cases used in this study, delusion was a consistent clinical feature. Whether the Acb tau accumulation is related to the psychiatric symptoms in TPD may be an issue for further investigation.

## Introduction

Tangle-predominant dementia (TPD), which is also referred to as neurofibrillary tangle predominant dementia, limbic neurofibrillary tangle dementia or senile dementia of the neurofibrillary tangle type, is a poorly understood and under-recognized tauopathy. TPD has been reported to comprise 0.7 to 5.8% of elderly patients with dementia [1-3]. TPD is characterized neuropathologically by numerous neurofibrillary tangles (NFT) in the limbic areas with no or occasional senile plaques throughout the brain. The clinical features of TPD include the late-adult onset, which is over 80 years in the majority of cases, and slow progression of dementia as compared with Alzheimer’s disease (AD). In patients with TPD, there is a propensity for the memory disturbance to be conspicuous with relative preservation of other cognitive functions. However, it is hard to distinguish TPD from AD on a clinical basis and, thus, diagnosis of TPD in most cases is only made postmortem.

The etiology of TPD is unknown. NFT in TPD consist of both 3-repeat (3R) and 4-repeat (4R) isoforms of hyperphosphorylated tau (tau), and the neuronal cell types bearing NFT in TPD are similar to those in AD. TPD seems to be a disorder that is related to AD, if it is not an atypical form of AD. TPD, as a subtype of tauopathy, is also included in the group described as neuropathologically-defined frontotemporal lobar degeneration [4,5]. In fact, cortical lesions in TPD are localized to the mediobasal temporal cortex. Thus, the situation of TPD in the groups of dementing neurodegenerative diseases remains unclear from both clinical and neuropathological points of view.

A neuropathological characteristic of TPD is the heavy accumulation of NFT in the hippocampal regions, with few or occasional NFT in neocortical areas beyond the collateral sulcus. Compared with AD patients in which a similar number of NFT occurs in the hippocampal regions, neuronal cell loss, tissue rarefaction and gliosis are less prominent in TPD, even in NFT rich areas. Changes in the neocortex are modest, with well-preserved laminar structures and unremarkable neuronal cell loss. The cortical expansion of NFT in TPD is considered to follow in principle the hierarchical pathway described in AD by Braak and Braak [6] but to be limited to stage IV. In the hippocampal regions, the density of NFT is higher than in AD [7] and ghost tangles are very frequent [3]. Tau pathology in the subcortical structures in TPD has not been well studied. The occurrence of NFT in the amygdala, the nucleus basalis of Meynert, the substantia nigra and the locus coeruleus, regions where NFT frequently occur in AD cases, have been reported in TPD [3,8].

**Figure 1:**
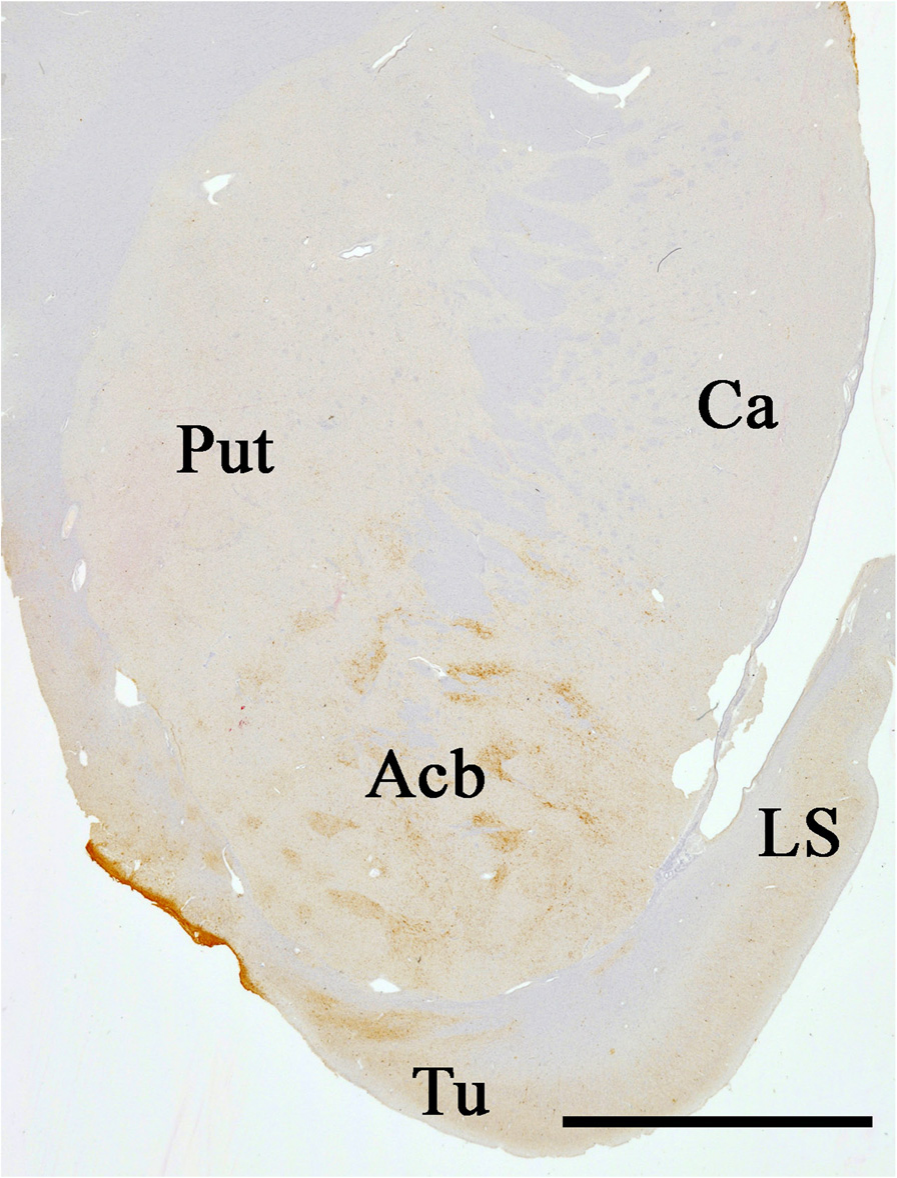
**A semi-macro photograph of the basal ganglia from a TPD case stained with AT8.** Faint immunoreaction is seen in the nucleus accumbens (Acb), lateral septal nucleus (LS) and the olfactory tubercle (Tu) even at this low power magnification. Ca: caudate nucleus; Put: putamen. Scale bar = 1 cm.

The nucleus accumbens (Acb) is located in the region where the caudate head and the rostral putamen meet near the septum pellucidum (Figure 1). The Acb and the olfactory tubercle form the ventral striatum in the forebrain. The Acb is a key component of the limbic striatal loop in which the Acb receives fibers from the prefrontal cortex, amygdala, hippocampus and ventral tegmental area (VTA) and projects to the ventral pallidum [9-12]. The ventral pallidum sends axonal projections to the dorsomedial thalamic nucleus, which then projects to the prefrontal cortex to close the loop [13,14]. The dopaminergic input from the VTA modulate the activity of this loop [15]. The Acb is considered to be involved in cognition, emotion and emotional behaviors such as pleasure, fear, aggression, addiction and reward [16,17]. The limbic striatal loop is, therefore, one of the major targets of studies on the pharmacological actions of anti-psychotic drugs [18,19].

In the present study, we found the frequent and consistent tau accumulation in the Acb in TPD. Tau positive neurons were also found in the Acb in some AD and on-demented aged subjects but much fewer in the majority of such cases. We speculate that the lesions in the Acb play a role in some psychiatric symptoms such as delusion, which is often conspicuous in TPD.

## Materials and methods

We used brain tissues, archived in our laboratory, from 7 patients with TPD, 22 with AD and 11 subjects without dementia or other neurological disease. The demography, Braak and Braak’s NFT stages and brain weight in each patient group are summarized in Table 1. Diagnoses were initially made on a clinical basis and were confirmed in every case by neuropathological examination. Clinical and neuropathological diagnoses of TPD followed the descriptions in previous articles [3,7,20]. Diagnoses of AD were made if the CERAD plaque score was ‘C’ [21] and the Braak and Braak’s NFT stage was IV or higher [6]. In TPD and AD cases with the NFT stage III or IV, Lewy body pathology was confirmed to be absent or mild/stage 1 [22] in the hippocampus, parahippocampal gyrus and temporal neocortex to exclude the possibility of Dementia with Lewy bodies. In all cases, the patient or, in any case where the patient had died, his/her next of kin gave the written consent for autopsy and postmortem analyses for research purposes. This study was approved by the ethics committee in the Tokyo Metropolitan Institute of Medical Science and was performed in accordance with the ethical standards laid down in the 1964 declaration of Helsinki and its later amendments.

**Table 1:** Summary of patient groups used in this study

	**TPD**		**AD**		**Non-demented**
**Braaks’ NFT stages**	**III or IV**	**IV**	**V**	**VI**	**I-III**
Number of cases	7	10	8	4	11
Gender (male/female)	1/6	6/4	4/4	2/2	7/4
Age at death*	88.4 ± 7.2	81.5 ± 8.5	86 ± 3.5	81.5 ± 8.8	81 ± 7.0
Disease duration (y)*	4.7 ± 2.9	6.1 ± 6.7	6.2 ± 3.5	6.6 ± 6.0	n/a
Brain weight (g)*	1,137 ± 135.3	1,134 ± 174.8	1,119 ± 77.2	1,008 ± 156.5	1,146 ± 77.1

*TPD*, Tangle-predominant dementia; *AD*, Alzheimer’s disease; *NFT*, neurofibrillary tangles.

*Data are shown as mean ± S.D.

For routine neuropathological examinations, formalin-fixed, paraffin-embedded brain blocks were cut into 10 μm thick sections and stained with hematoxylin and eosin (HE), Klüver-Barrera, modified Gallyas-Braak and methenamine silver staining. Tissue sections of the mediobasal temporal cortex containing the hippocampus, entorhinal cortex and temporal neocortex were stained for tau and amyloid β protein (Aβ) by immunohistochemistry. Sections of the rostral striatum with the Acb and the septal nuclei were stained for tau. In TPD cases, additional tau immunohistochemistry was performed for the nucleus basalis of Meynert, amygdala and substantia nigra. The hippocampus, parahippocampal gyrus and adjacent temporal neocortex were also stained for phosphorylated α-synuclein and phosphorylated TDP-43 in TPD cases.

For more detailed immunohistochemical analyses, small blocks of brain the tissues were dissected at autopsy and fixed in 4% paraformaldehyde (PFA) for 2 days. The cryocut sections of 30 μm thickness were used for the high sensitive, free-floating immunhistochemical staining [23]. The antibodies used in this study are listed in Additional file 1: Table S1. The primary antibody labeling was visualized with 3,3′-diaminobenzidine as a chromogen, in combination with the Envision Plus® kit (Dako Japan, Tokyo). For enhanced thioflavin-S staining, tissue sections were pretreated with KMnO_4_ for 20 min and, subsequently, with sodium borohydride for 4 min [24]. Sections were then stained with 0.05% thioflavin-S in 50% ethanol in the dark for 8 min, followed by differentiation in two changes of 80% ethanol for 10 sec each time and three washes in large volumes of distilled water. Following incubation in a high salt solution containing 411 mM NaCl, 8.1 mM KCl, 30 mM Na_2_HPO_4_ and 5.2 mM KH_2_PO_4_, pH 7.2 at 4°C for 30 min, sections were briefly rinsed with distilled water and observed by fluorescence microscopy.

For immunoelectron microscopy, both post-embedded and pre-embedded procedures were used. For the former, the 4% PFA-fixed small tissues were embedded in LR White Resin® (London Resin, U.K.) without further fixation. The ultra-thin sections were stained with AT8, which was followed by incubation with anti-mouse IgG conjugated with 10 or 20 nm gold colloidal particles (BBinternational, U.K.). For the pre-embedding procedure, the 4% PFA-fixed free-floating sections were stained with AT8 in combination with Alexa Fluor 488 FluoroNanogold anti-mouse IgG (Nanoprobes, U.S.A.). Following examination by fluorescence microscopy to localize the positive labeling, the sections were postfixed with 2% glutaraldehyde and then treated with HQ Silver Enhancement Kit (Nanoprobes, U.S.A.). After the treatment with 1% osmium tetroxide, which was followed by 2% uranyl acetate, the sections were embedded in epoxy resin (Querol 812, Nissin EM, Japan). Ultrathin sections were cut and observed by a transmission electron microscope (JEM-1400, JEOL, Japan).

For immunoblot analyses, fresh frozen samples of the Acb and the parahippocampal cortex were obtained from 3 TPD cases (cases 3, 4 and 6) and 3 AD cases. The Braak and Braak’s NFT stages of the AD cases were 4, 5 and 6, respectively. Brain tissue was homogenized in 2 vol of TS buffer (50 mM Tris–HCl, 150 mM NaCl, pH 7.5), with a mixture of protease inhibitors and centrifuged at 200,000 g for 20 minutes at 4°C. The supernatant was taken as the soluble fraction and the pellet was used to further extract the sarkosyl-insoluble fraction as described previously [25]. Dephosphorylation of the sarkosyl-insoluble fractions was performed by incubation of the samples with Escherichia coli alkaline phosphatase (type III, Sigma) as described previously [25]. HT7, a pan-tau monoclonal antibody (Additional file 1: Table S1), was used for immunoblotting. Primary antibody labeling on the membranes was visualized with 3,3′-diaminobenzidine as a chromogen, in combination with a Vectastain ABC kit® (Vector Lab., USA).

For semiquantitative analyses of immunohistochemically stained tissue sections, the density of AT8 positive tau accumulation was graded to be 0 for absent, 1 for low, 2 for intermediate and 3 for high, based on microscopic observations at ×200 magnification. The Acb, septal nuclei, caudate nucleus, hippocampal CA1, entorhinal cortex and temporal neocortex were assessed in TPD, AD and non-demented aged subjects. The Mann–Whitney *U* test was used for statistical analyses using Graph Pad Prism 4 software (Graph Pad Software, U.S.A.).

## Results

### TPD cases used in the present study

The demographic, pathologic, and clinical information of the TPD cases used in the present study is summarized in Tables 1 and 2. In general, both the clinical and neuropathological features are similar to those described in previous reports [1-3,7,26,27]. The average age at death is higher than that in AD. Moderate dementia was noted in 5 of the 7 cases but the other two were diagnosed as having mild cognitive impairment. Delusion was evident in 6 cases. Brain atrophy was mild, if present, and senile plaques were either absent or rare. Lacunar infarcts were seen in the globus pallidus in 2 cases. In all cases, heavy tau accumulation was seen in the limbic regions in the forms of NFT, diffuse cytoplasmic accumulations and neuropil threads. Tau accumulation was heavier in the subiculum and the CA1 region than in the entorhinal and transentorhinal cortices. Tau was also deposited in the amygdala, the septal nuclei and the basal nucleus of Meynert, and, less frequently, in the caudate nucleus and substantia nigra. A small amount of tau was found in the temporal neocortex but only in 3 cases. Such limbic-predominant distribution of tau pathology is consistent with previous reports [1,2,26,28]. A small number of argyrophilic grains were present in 2 cases.

**Table 2:** Demography and basic clinical and neuropathological features of TPD cases

	**Case 1**	**Case 2**	**Case 3**	**Case 4**	**Case 5**	**Case 6**	**Case 7**
Age at death	89	102	90	85	89	78	86
Sex	F	F	F	F	F	M	F
Dementia	+	+	+	+	MCI	MCI	+
*Psychiatric symptoms*							
Delusion	+	+	+	+	+	+	-
Anxiety	+	-	-	-	-	-	-
Depression	-	-	-	+	-	-	-
Brain weight (g)	940	970	1170	1300	1230	1220	1130
Atrophy	mi(Fr)	mi(Fr/T)	-	-	-	-	-
Plaque stage (1)	0	0	A*	0	0	0	0
NFT stage (1)	III	III	III	III	IV	III	III
Argyrophilic grain stage (2)	0	0	0	0	II	II	0
Hippocampal sclerosis	-	-	+	-	-	-	-
Vascular lesions	+	-	-	+	-	-	-
α-synuclein (hip/T**)	+§	-	-	-	-	-	-
TDP-43 (hip/T**)	-	-	-	-	-	-	-
Acb tau score	3	2	2	3	3	2	3

*F*, Female; *M*, Male; *MCI*, Mild cognitive impairment; *mi*, Mild; *Fr*, Frontal; *T*, Temporal; *Acb*, The nucleus accumbens. *A small number of diffuse Aβ deposits were seen in the temporal cortex. The Acb tau score was determined according to the method described in the text. (1) The senile plaque and NFT staging were based on the description by Braak and Braak [6]. (2) The argyrophilic grain staging was based on the description by Saito et al. [29]. **Immunohistochemistry for α-synuclein and TDP-43 was performed in tissue sections of the hippocampus, parahippocampal gyrus and adjacent temporal neocortex. § α-Synuclein pathology in this case was mild, corresponding to stage 1 by the 3^rd^ report of the DLB consortium [22].

### Tau accumulation in the Acb in TPD

**Figure 2:**
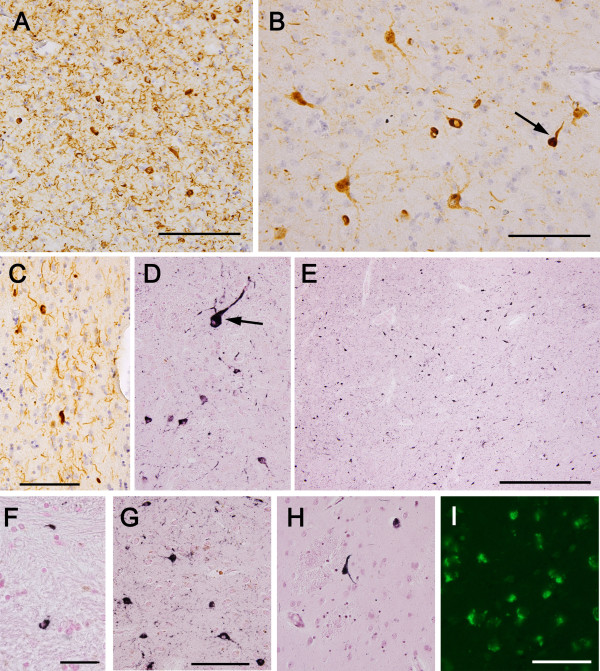
**Tau accumulation in the Acb in TPD. A** through **G** are immunohistochemistry with phosphorylation or conformational change specific tau antibodies. **A**, **B**, **C** and **I**: 4% paraformaldehyde-fixed, frozen-cut, 30 μm thick Sections. **D** through **H** are formalin-fixed, 10 μm thick, paraffin Sections. **A**: immunohistochemistry with AP422. Tau positive neurons are associated with many neuropil threads. Scale bars = 200 μm. **B**: immunohistochemistry with PHF-1. The majority of tau positive neurons show pretangle-like, diffuse or granular cytoplasmic labeling. Among them, apparent NFT are also seen but less frequently (arrow). Scale bar = 100 μm. **C**: immunohistochemistry with MC1, a conformational change specific antibody. Scale bar = 100 μm. **D** through **G** are immunohistochemistry with AT8. **D**: the vast majority of tau positive neurons are of medium-size but, occasionally, large neurons are also stained positively for tau (arrow). At the same magnification as **C. E**: tau positive neurons are not evenly distributed in the Acb. Scale bar = 500 μm. **F**: a glial coiled body. Scale bar = 25 μm. **G** and **H**: the nearby sections from the same case with AT8 immunohistochemistry **(G)** and Gallyas-Braak staining **(H)**.** I**: thioflavin S staining reveals granular cytoplasmic labeling of neurons. Scale bar = 100 μm.

In addition to the previously reported tau distribution, we found a considerable number of tau positive neurons in the Acb in all TPD cases used in this study (Figures 1 and 2). Similarly to the hippocampus, numerous neuropil threads were associated with tau positive neurons (Figure 2A). The tau positive neurons and neuropil threads were labeled with all the anti-tau antibodies used in the present study (Figures 2A-D). They included conformational change-specific, phosphorylation-specific and phosphorylation-independent antibodies (Additional file 1: Table S1). The staining pattern varied, which partly depends on the affinity of the antibody and the localization of the antigen epitope recognized by each antibody. Preservation of the epitope in tissue sections is affected by aggregation, degradation and post-mortem processing such as fixation. The majority of tau positive neurons in the Acb showed pretangle-like, diffuse or granular accumulation of tau in the cytoplasm (Figures 2B). Flame-like NFT, the common form in the hippocampus in TPD, were also present but not frequent (Figure 2B, arrow). The vast majority of tau positive neurons were medium sized but, occasionally, large neurons were also stained positively for tau (Figure 2D, arrow). Tau positive neurons and threads were not distributed evenly in the Acb. Rather, areas with sparse- and dense-tau pathology were intermingled (Figure 2E). Occasional glial coiled bodies were seen in the majoriy, if not all, of the cases (Figure 2F). Occurrence of glial coiled bodies in other brain regions in TPD has been reported previously [3]. Gallyas-Braak staining labeled only a small number of NFT in the Acb, while tau immunohistochemistry of nearby sections from the same patient revealed many positive cells (Figure 2G and 2H). Enhanced thioflavin-S staining labeled many neurons (Figure 2I).

The density of tau positive neurons and neuropil threads varied somewhat among the TPD cases. In TPD, no clear association was seen between the degree of Acb tau pathology and the Braak and Braak’s NFT stage or the presence or absence of Aβ deposits, argyrophilic grains [29] and vascular lesions (Table 2). Despite the consistent tau accumulation in the Acb in TPD, we were not able to find severe neuronal loss or gliosis by HE staining.

**Figure 3:**
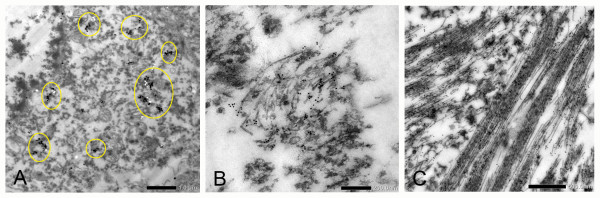
**Immunoelectron microscopy of TPD brain with AT8 and immunogold labeling. A**: in the Acb, the immunogold labeling in neurons was mostly localized to the granular structures (indicated by circles). Paired-helical filaments (PHF) were rare. Scale bar = 1.0 μm. **B**: sparse bundles of PHF were scattered in the neuronal cytoplasm. Scale bar = 200 nm. **C**: in the hippocampal CA1 region, prominent bundles of AT8 positive PHF were seen. Scale bar = 500 nm.

Immunoelectron microscopy of the Acb in TPD with a tau antibody, AT8, revealed positive labeling of granular structures in the neurons (Figure 3A). Small and sparse bundles of short filamentous structures were occasionally seen to be stained positively for AT8 in the neuronal cytoplasm and neuropil (Figure 3B). Some of them showed morphology consistent with paired helical filaments (PHF). Thus, the ultrastructure of tau accumulation in the Acb was different from that in the hippocampal CA1 region, where dense and long bundles of PHF were frequent and intensely labeled for AT8 (Figure 3C).

### Tau pathology in the Acb in AD and non-demented aged subjects

**Figure 4:**
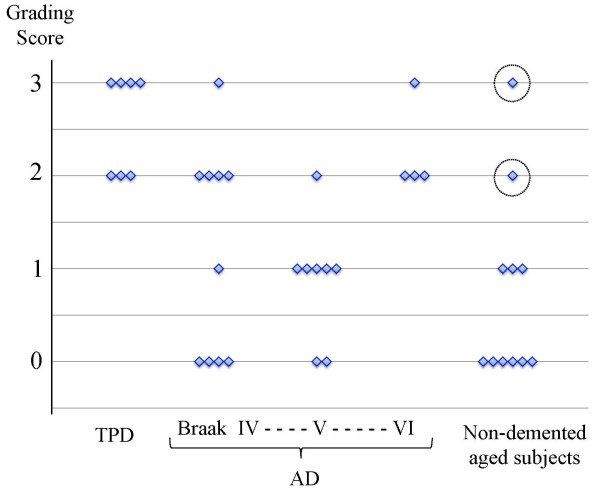
**A graph of the density of neuronal tau accumulation in the Acb.** The density of tau accumulation was graded as being 0 for absent, 1 for low, 2 for intermediate and 3 for high. Statistically significant differences were seen between TPD cases and non-demented, aged subjects (*P =* 0.0031) as well as AD cases in NFT stage IV (*P =* 0.0192) and V (*P =* 0.0022). The points encircled by a broken line in the non-demented, aged group indicate that the cases were over age 90.

We then investigated the Acb in AD and non-demented, aged subjects. Tau positive neurons were found in some, but not all, AD patients and non-demented, aged subjects (Additional file 2: Figure S1A). In these groups, however, only a limited number of cases showed tau pathology which was similarly abundant to that in TPD (Figure 4). In AD patients with heavy tau accumulation in the Acb, the caudate nucleus was also affected, a feature which distinguished AD from TPD. In TPD, the caudate tau lesions were either absent or, if present, very mild in all cases. In addition, senile plaques with tau positive dystrophic neurites were scattered in the Acb of such AD cases (Additional file 2: Figure S1B). In AD cases with mild tau pathology in the Acb, large neurons preferentially contained tau, a finding which was similar to the caudate nucleus in AD. In AD cases with heavy tau pathology in the Acb, such large neuron predominance became unclear and many tau positive, medium-sized neurons were seen. In both AD and non-demented, aged subjects, neuropil threads were also present in those with tau positive neurons in the Acb (Additional file 2: Figure S1C). The form of tau accumulation in AD patients and non-demented, aged subjects was similar to that in TPD, being predominantly pre-tangle like, diffuse accumulation in the cytoplasm.

The density of neuronal tau accumulation was graded to be 0 (absent) through 3(high) in AT8 immunostained tissue sections. Figure 4 illustrates the results in the Acb. Tau density in the Acb in the AD group was highly variable, except that the cases in Braak and Braak’s NFT stage VI were either grade 2 or 3. Statistically significant differences were seen between the TPD cases and the non-demented, aged subjects (*P =* 0.0031) as well as the AD cases with NFT stage IV (*P =* 0.0192) and those with NFT stage V (*P =* 0.0022). Two non-demented, aged subjects with tau accumulation in the Acb were both over age 90. These 2 cases, similarly to TPD, lacked tau accumulation in the caudate nucleus and showed more NFT in the subiculum than in the entorhinal cortex. The results of semiquantitative analyses confirmed our observation that tau accumulation in the Acb was a remarkable finding in TPD. We performed similar analyses for a number of brain regions. The results are summarized in Table 3 as the averages of the graded scores for tau accumulation in each group. The concentration of tau pathology in the limbic structures, including the Acb and septal nuclei, in TPD contrasted with the broad distribution over the neocortex in AD.

**Table 3:** Summary of the semiquantitative grading of tau accumulation

	**Braak stage**	**No. of cases**	**Acb**	**Caudate nucleus**	**Septal nucleus**	**CA1**	**Ent**	**Temp**
TPD	III-IV	7	2.6	0.7	2.2	3	2.57	0.4
Non-demented	I-II	3	0	0	0	0.67	1	0
	III	8	1.13	0.25	0.63	1.38	2	1
AD	IV	10	1.2	0.6	1.4	2.67	3	1.4
	V	8	0.9	0.9	1.3	2.89	2.9	2.5
	VI	4	1.3	1.3	1.7	3	3	3

The numbers indicate averages of the scores in each group. The degree of tau pathology was qualitatively scored as 0: absent 1: low 2: intermediate 3: high.

*TPD*, Tangle predominant dementia; *AD*, Alzheimer’s disease; *NFT*, Neurofibrillary tangles; *Acb*, nucl. Accumbens; *CA1*, Hippocampal CA1 region; *Ent*, Entorhinal cortex; *Tmep*, Temporal neocortex.

### Immunoblot analyses

**Figure 5:**
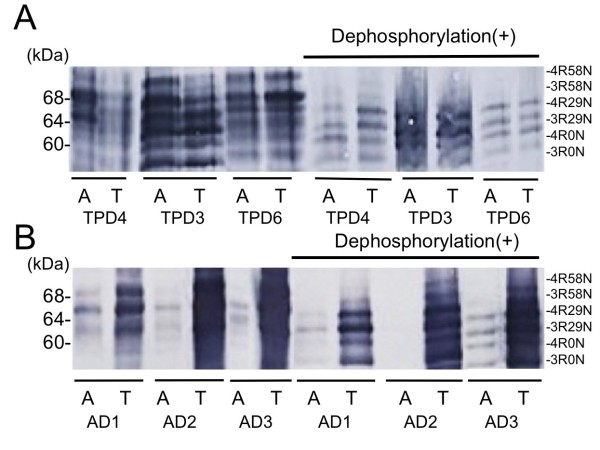
**Immunoblot analyses of the sarkosyl insoluble tau.** The sarkosyl insoluble fractions of the Acb (A) and the parahippocampal cortex (T) from TPD **(A)** and AD **(B)** were analyzed by immunoblot. A pan-tau antibody, HT7, was used. The Acb samples show the 3R + 4R isoform pattern similar to that in the parahippocampal cortices in both TPD and AD.

The results of immunoblot analyses of samples from TPD and AD patients are shown in Figure 5. The tau band patterns in the sarkosyl insoluble fraction appeared to be essentially the same between TPD and AD, while the amount of insoluble tau was far smaller in the Acb than in the parahippocampal cortex in AD. It has to be noted that, because of the very high concentrations of insoluble tau in the parahippocampal cortex samples, the amounts of samples applied to the gels had to be reduced in AD cases. This resulted in the relatively weak signals for the Acb samples in AD cases. The dephosphorylated samples of the Acb and parahippocampal cortex showed the 3R + 4R isoform pattern in both TPD and AD.

### The Acb tau pathology and the presence/absence of clinical history of delusion

Finally, we examined if the degree of the Acb tau pathology was different between the subjects groups with and without the history of delusion in the clinical records (Additional file 3: Figure S2). In the group of subjects with Braaks’ NFT stages III and IV, which included NFT stage III non-demented aged subjects, all TPD cases and NFT stage IV AD cases, the Acb tau score was higher in those with clinical history of delusion than those without it (*p =* 0.033). Similarly, the less conspicuous but still significant difference was seen in the group of all NFT stage AD cases (*p =* 0.049).

## Discussions

There is significant overlap in the distribution of NFT between TPD and AD. However, an early genetic study of TPD cases indicated a paucity of the apolipoprotein E ϵ4 allele, which currently is the most powerful risk factor for AD [30]. More recently, a report has been made on the significant associations of TPD with the *MAPT* H1 haplotype as well as with some polymorphisms within the region of *MAPT* encoding the 3′ UTR [31]. Thus, together with the striking paucity of Aβ deposition, it seems that TPD is a unique neuropathological entity that has to be studied separately from AD.

The clinical and neuropathological features of the TPD patients we used in the present study generally agreed with those described in preceding articles [3,32]. In addition to the already well-known distribution of tau pathology, we found a considerable number of tau positive neurons and neuropil threads in the Acb. The Acb consists of the predominant medium-sized neurons and the occasional large neurons. Both cell types were affected by tau pathology in TPD. This contrasted AD with mild Acb tau pathology, in which large neurons were affected preferentially. Such a result is consistent with the previous reports which described that large neurons are more vulnerable in AD [33] and prone to tau accumulation [34]. In AD cases with the heavy Acb tau pathology, many medium-sized neurons were also tau positive. In the absence of TPD cases with the mild Acb tau pathology, it remains to be determined whether the difference is attributable to the distinct pathomechanisms between AD and TPD or to the variable vulnerability of different neuronal cell types.

The Acb may not be a region which is routinely sampled in a number of laboratories. Further, tau pathology in the Acb is not well stained by the Gallyas-Braak method. These facts together might explain the absence of previous reports on the occurrence of tau lesions in the Acb. The mechanism by which the Gallyas-Braak staining labels NFT remains to be determined. In TPD, insoluble tau consists of a mixture of the 3R and 4R isoforms in both the Acb and hippocampus, but NFT are intensely labeled by Gallyas-Braak staining in the latter. It may be noteworthy that, in the Acb, tau pathology occurs in a form of diffuse or granular cytoplasmic accumulations in the majority of tau positive neurons and that, ultrastructurally, PHF were rare. These contrasted with the hippocampal lesions where dense bundles of PHF were frequently seen. Obviously, factors that affect the reactivity of abnormal tau deposits to Gallyas-Braak staining need further clarification.

Tau pathology is considered to propagate in the brain from an affected region to another along the fiber connections, by spreading through the neuropil, or by both. A proposed mechanism for such propagation is a prion-like, seed-dependent conformational change and subsequent aggregation of the molecule, with a breakdown of the aggregate that generates the next seeds. In such a manner, the tau isoform pattern in the initial aggregates may be maintained in the later aggregate formations [35]. In the present study, we found that the Acb lesion in TPD was 3R + 4R tauopathy, a result which suggests the common origin of the tau pathology in the Acb with that in the hippocampus. The prevalence of diffuse cytoplasmic accumulations suggests that tau pathology in the Acb occurs later in the disease progression. In the hippocampus in TPD, many ghost tangles are seen, suggesting that the hippocampal lesions precede the Acb lesions.

Occurrence of NFT in the Acb in AD was reported previously [36,37]. In the present study, however, we have found that tau accumulation in TPD is more frequent and consistent than AD. The Acb receives direct and massive projections from the hippocampal CA1 and subiculum [14,38,39]. It has been repeatedly reported in TPD that the density of NFT is higher in the hippocampal CA1 and subiculum than in the entorhinal cortex [1,2,7,28]. Thus, the heavy tau pathology in the subiculum and CA1, through neural circuit-mediated propagation to the Acb, may result in the more pronounced tau accumulation in the Acb in TPD than in AD. Such an idea may be consistent with our finding that the difference in Acb tau pathology was statistically significant between TPD and AD in NFT stages IV and V but not in AD at stage VI. In AD with Acb lesions, tau accumulation was also found more frequently in the caudate nucleus than was the case in TPD. The caudate nucleus receives massive innervations from the cerebral cortex where, unlike TPD, tau pathology is severe in AD. On the other hand, the septal nuclei, like the Acb, receive direct projections from the subiculum and CA1 [14,38]. Again, we found, in the present study, heavier tau accumulation in these areas in TPD than in AD at NFT stages IV and V.

TPD is primarily an amnestic disease with relatively mild non-amnestic symptoms of dementia. In 6 of 7 TPD cases used in this study, however, delusion was a consistent clinical feature. This may be partly attributable to the fact that our brain tissue archive is principally based on the psychiatric hospital autopsies. However, occurrence of psychiatric symptoms has also been described in a number of previous reports on TPD. As an example, Jellinger et al. reported depression in 17.5% and paranoid ideas in 15% of TPD cases [3]. The Acb is part of the mesolimbic system in which the Acb receives dopaminergic input from the VTA. Recent evidence suggests that, in schizophrenia, functional abnormality in the Acb causes excessive release of dopamine from the VTA, which then results in the psychiatric symptoms [19,40-43]. While neuronal loss was not apparent in the Acb in TPD, it may be noteworthy that association of intraneuronal tau aggregation with clinical symptoms has been suggested in early stage AD lesions [44]. In AD, cases with more neocortical NFT were reported to be associated with more psychosis [45]. Thus, tau accumulation in the Acb could be related to the frequent delusion in TPD. Delusion and other psychotic symptoms may occur by multiple mechanisms in dementia patients. We have to note that 2 of the 7 TPD cases had argyrophilic grain pathology and that psychotic symptoms are known to be common in the patients with argyrophilic grain disease [46]. Whether the Acb tau accumulation is related to the psychiatric symptoms in TPD may be an issue for further investigation.

In the present study, we found that tau pathology occurred unevenly in the Acb in TPD (Figures 1 and 2E). The striatum is not uniform and has distinct neurochemical compositions and connections that are referred to as matrix and striosomes. The similar but more complex compartmentation was reported in the human Acb [47]. We have performed additional immunohistochemistry for tyrosine hydroxylase (TH) and tau in serially-cut, free-floating sections in two TPD cases, in which the remnants of Acb blocks were available after the initial sectioning for the main body of this study. Comparison of the adjacent sections stained for TH and tau indicates that tau pathology preferentially occurs in areas where the fine, mesh-like TH staining is relatively light (Additional file 4: Figure S3). Such a result suggests the relationship between the uneven distribution of tau pathology and neurochemical heterogeneity in the Acb. However, because of the limited number of currently available samples and of the more complex neurochemical architecture in the Acb than the simple matrix-striosome structure in the caudate nucleus [47], future, extensive studies should be needed for further exploration.

## Conclusions

We have found frequent tau accumulation in the Acb in patients with TPD. Both the medium-sized and large neurons are affected. While similar tau accumulation was seen in a small number of all AD patients, it was far more frequent and consistent in TPD than AD. The tau isoforms abnormally accumulated in the Acb were 3R and 4R, which suggests a common origin with the hippocampal tau pathology. The Acb receives direct and massive projections from the hippocampal CA1 and subiculum where tau pathology is extremely severe in TPD. Such a result may support the idea that abnormal tau aggregation propagates via neural circuits. Tau accumulation in TPD should be a subject of further investigations to approach the long-lasting issue of the simultaneous deposition of Aβ and tau in AD. In addition, the relationship between the tau pathology in the Acb and such psychiatric symptoms as delusion in TPD needs further exploration.

## Abbreviations

(TPD): Tangle-predominant dementia; (NFT): Neurofibrillary tangles; (AD): Alzheimer’s disease; (3R): 3-repeat; (4R): 4-repeat; (Acb): Nucleus accumbens; (VTA): Ventral tegmental area; (HE): Hematoxylin and eosin; (PFA): Paraformaldehyde; (PHF): Paired helical filaments; (Aβ): Amyloid β protein; (TH): tyrosine hydroxylase.

## Competing interests

The authors declare that they have no competing interests.

## Authors’ contributions

IK carried out the microscopic observation, immunoblot and statistical analyses. IK also drafted the initial manuscript. MHa conducted the sample preparation and immunoblot and carried them out with IK. TA participated in the design and coordination of the study. KI carried out the microscopic observation with IK. KO, KN and NA organized the brain archives including clinical information, selected appropriate cases, and performed neuropathological analyses of all cases used in this study. OK participated in the design of the study and performed statistical analyses with IK. SH conceived of the study and participated in the initial design. MHo contributed to the reagents, materials and analysis tools, and conducted free-floating immunohistochemistry. YH participated in the design of the study and helped to draft the manuscript. HA supervised the design and coordination of the study and worked up the manuscript. All authors read and approved the final manuscript.

## Authors’ information

IK is a graduate student of Department of Psychiatry, Graduate school of Medicine, Yokohama City University School of Medicine. MHa is the senior director of Department of Neuropathology and Cell Biology, Tokyo Metropolitan Institute of Medical Science (TMIMS). TA, KI, NA and SH contributed to this study as visiting scientists of Dementia project, TMIMS. TA is also an associated professor of Department of Psychiatry, Graduate School of Comprehensive Human Sciences, University of Tsukuba. KO and KN are psychiatrists and neuropathologists in Tokyo Metropolitan Matsuzawa Hospital (TMMH), visiting scientists of Dementia project, TMIMS and in charge of the brain archive of TMMH/TMIMS. OK is an associate professor of Department of Psychiatry, Graduate school of Medicine, Yokohama City University School of Medicine. MHo is a chief researcher of Dementia project, TMIMS. YH is a professor of Department of Psychiatry, Graduate school of Medicine, Yokohama City University School of Medicine. HA is the senior director of Dementia project, TMIMS.

## Supplementary Material

Additional file 1: Table S1 The primary antibodies used in this study. Click here for file

Additional file 2: Figure S1 Tau accumulation in the Acb in AD and non-demented, aged subjects. Immunohistochemistry with AT8. A: absence of tau positive neurons in an AD case in Braak and Braak’s NFT stage IV. Scale bar = 100 μm in A-C. B: a diffuse cytoplasmic staining, neuropil threads and duystrophic neurite in a senile plaque in an AD case in NFT stage VI. C: a tau positive neuron and neuropil threads in a non-demented, aged subject. Click here for file

Additional file 3: Figure S2 A graph of the density of neuronal tau accumulation in the Acb. The left plots: the group of Braaks’ NFT stages III and IV, which includes non-demented aged subjects, TPD cases and AD cases with Braaks’ NFT stage IV. Cases with delusion in the clinical history show higher tau score than those without delusion in the Acb. The right plots: the group of AD cases with Braaks’ NFT stages IV through VI. Again, cases with delusion show higher tau score than those without delusion. Click here for file

Additional file 4: Figure S3 The serial section immunohistochemistry for tau and tyrosine hydroxylase (TH). Forty micrometer thick, free floating sections were cut serially from two tangle predominant dementia (TPD) cases, in which the remnants of Acb blocks were available after the initial sectioning for the main body of the study. A set of every other section was stained for TH and the other set for tau with AT8. A and C: AT8 staining in a TPD case 1. B: TH staining of the section between A and C. In B, two types of areas are distinguished based on the modest difference in the density of fine, mesh-like TH staining. There is a propensity that tau pathology preferentially occurs in areas where the fine, mesh-like TH staining is relatively light (A, C). Scale bar = 2 mm in A (A, B and C are at the same magnification). D: higher power photomicrographs of the boxed areas in B and C. The left half is the staining with AT8 and the right half staining for TH. Scale bar = 400 micro-m (D). Click here for file
