# Supplementary material for: Tau accumulation in the nucleus accumbens in tangle-predominant dementia
Source: Acta Neuropathol Commun. 2014 Apr 8;2:40. doi: 10.1186/2051-5960-2-40 (PMC4023632; doi:10.1186/2051-5960-2-40)
Supplement: Additional file 1: Table S1 — The primary antibodies used in this study. [file 2051-5960-2-40-S1.docx]

**Additional file 1: Table S1**

The primary antibodies used in this study

| Antibody | Type | Source | Specificity |
| --- | --- | --- | --- |
| HT7 | Mouse monoclonal | Innogenetics | tau (aa 159-163) |
| A0024 | Rabbit polyclonal | Dako | tau C-terminal (aa 243-441) |
| MC-1 | Mouse monoclonal | Generous gift of Dr. Peter Davies | tau N-terminal (aa 2-10), conformation change specific |
| AT8 | Mouse monoclonal | Innogenetics | p-tau (pSer199 & pSer202) |
| PHF-1 | Mouse monoclonal | Generous gift of Dr. Peter Davies | p-tau (pSer396 & pSer404) |
| AP422 | Rabbit polyclonal | Made by M. Hasegawa | p-tau (pSer422) |
| Aβ (E50) | Rabbit polyclonal | Made by H. Akiyama | Aβ (aa17-31) |
| pα-synuclein | Mouse monoclonal | Wako | α-synuclein (pSer129) |
| pTDP-43 | Rabbit polyclonal | Made by M. Hasegawa | TDP-43(pSer409/410) |
| Tyrosine hydroxylase | Mouse monoclonal | Millipore (Chemicon) | Tyrosine hydroxylase |
