# Supplementary figures and images for: Tau accumulation in the nucleus accumbens in tangle-predominant dementia
Source: Acta Neuropathol Commun. 2014 Apr 8;2:40. doi: 10.1186/2051-5960-2-40 (PMC4023632; doi:10.1186/2051-5960-2-40)

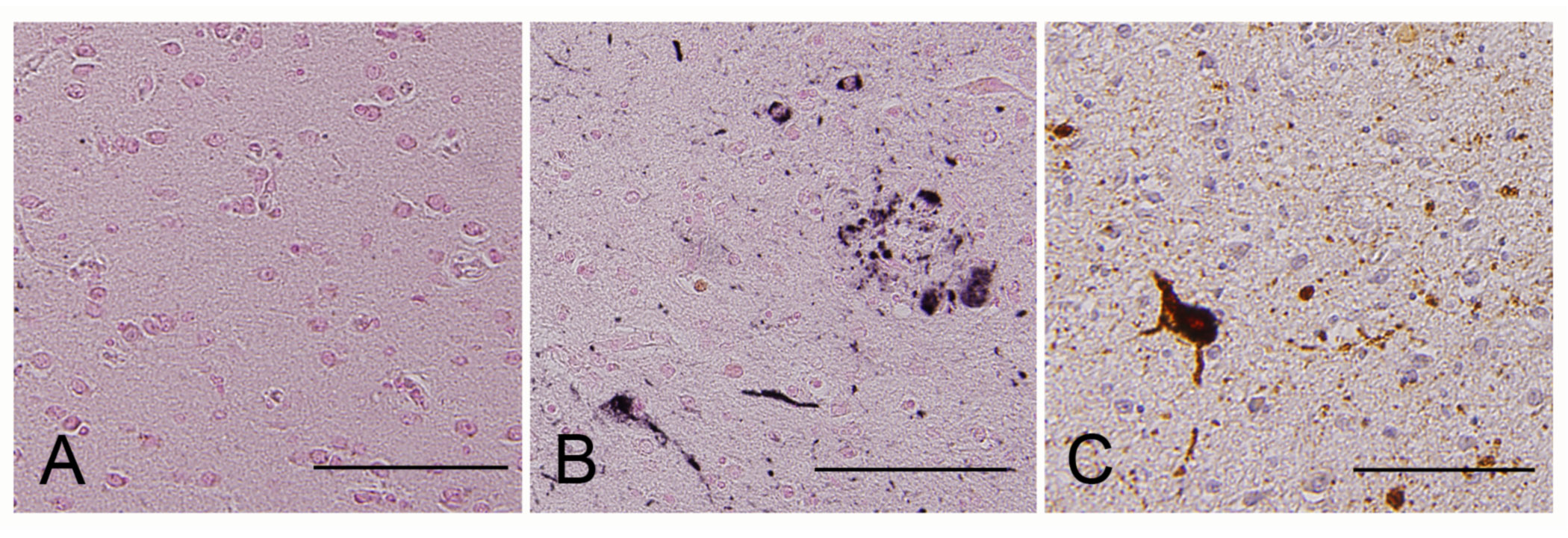

Supplement: Additional file 2: Figure S1 — Tau accumulation in the Acb in AD and non-demented, aged subjects. Immunohistochemistry with AT8. A: absence of tau positive neurons in an AD case in Braak and Braak’s NFT stage IV. Scale bar = 100 μm in A-C. B: a diffuse cytoplasmic staining, neuropil threads and duystrophic neurite in a senile plaque in an AD case in NFT stage VI. C: a tau positive neuron and neuropil threads in a non-demented, aged subject. [file 2051-5960-2-40-S2.jpeg]

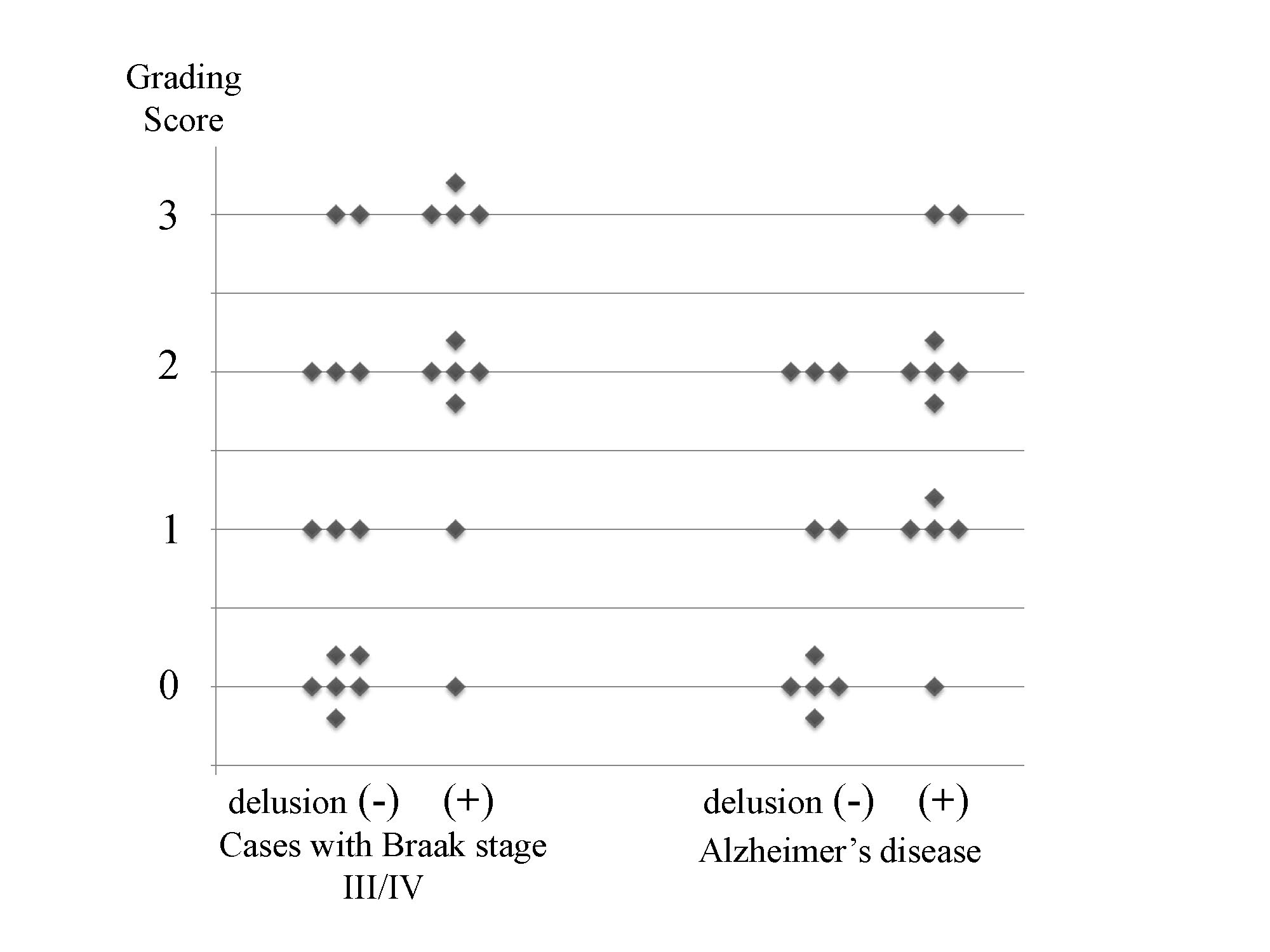

Supplement: Additional file 3: Figure S2 — A graph of the density of neuronal tau accumulation in the Acb. The left plots: the group of Braaks’ NFT stages III and IV, which includes non-demented aged subjects, TPD cases and AD cases with Braaks’ NFT stage IV. Cases with delusion in the clinical history show higher tau score than those without delusion in the Acb. The right plots: the group of AD cases with Braaks’ NFT stages IV through VI. Again, cases with delusion show higher tau score than those without delusion. [file 2051-5960-2-40-S3.jpeg]

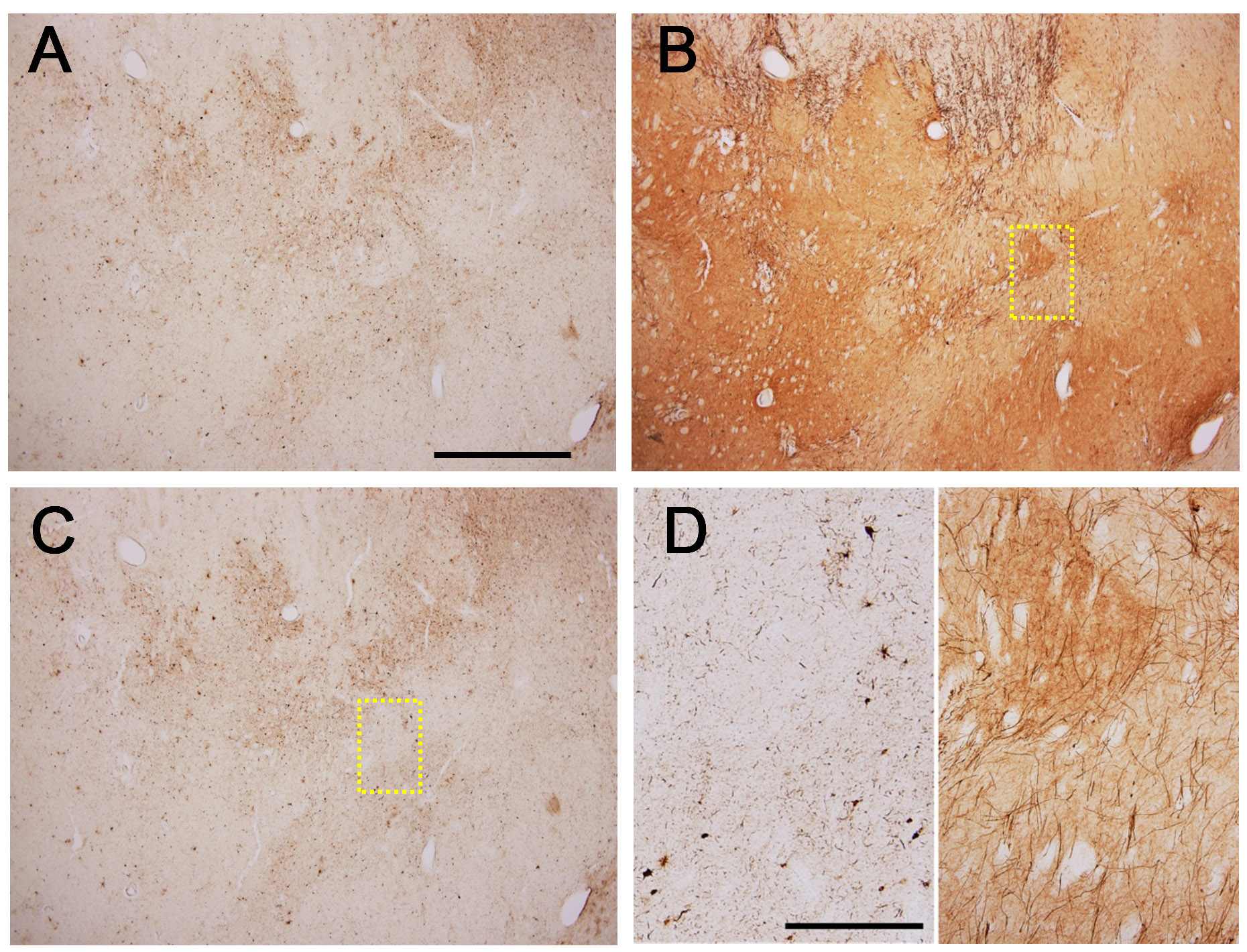

Supplement: Additional file 4: Figure S3 — The serial section immunohistochemistry for tau and tyrosine hydroxylase (TH). Forty micrometer thick, free floating sections were cut serially from two tangle predominant dementia (TPD) cases, in which the remnants of Acb blocks were available after the initial sectioning for the main body of the study. A set of every other section was stained for TH and the other set for tau with AT8. A and C: AT8 staining in a TPD case 1. B: TH staining of the section between A and C. In B, two types of areas are distinguished based on the modest difference in the density of fine, mesh-like TH staining. There is a propensity that tau pathology preferentially occurs in areas where the fine, mesh-like TH staining is relatively light (A, C). Scale bar = 2 mm in A (A, B and C are at the same magnification). D: higher power photomicrographs of the boxed areas in B and C. The left half is the staining with AT8 and the right half staining for TH. Scale bar = 400 micro-m (D). [file 2051-5960-2-40-S4.jpeg]
